# Impact of Comorbidities on the Long-Term Survival Rate of Patients Aged 60 Years and Older Undergoing Deceased Donor Kidney Transplantation Versus Continued Waitlisting

**DOI:** 10.3390/jcm15062378

**Published:** 2026-03-20

**Authors:** Jae Jun Lee, Jin-Myung Kim, Hye Eun Kwon, Young Hoon Kim, Youngmin Ko, Sung Shin, Joo Hee Jung, Chung Hee Baek, Hyosang Kim, Hyunwook Kwon

**Affiliations:** 1 Department of General Surgery, Kangbuk Samsung Hospital, Sungkyunkwan University School of Medicine, Seoul 06351, Republic of Korea; jaejun8.lee@samsung.com; 2 Division of Kidney and Pancreas Transplantation, Department of Surgery, Asan Medical Center, University of Ulsan College of Medicine, Seoul 05505, Republic of Korea; jinmyung.kim16@amc.seoul.kr (J.-M.K.); rleh1104@amc.seoul.kr (H.E.K.); bch393@naver.com (C.H.B.); 3 Division of Nephrology, Department of Internal Medicine, Asan Medical Center, University of Ulsan College of Medicine, Seoul 05505, Republic of Korea; mateus@amc.seoul.kr

**Keywords:** kidney transplantation, renal dialysis, aged, cardiovascular diseases

## Abstract

**Background:** The survival benefits of kidney transplantation (KT) versus dialysis in elderly end-stage renal disease (ESRD) patients, particularly those with cardiovascular disease (CVD), remain uncertain. **Methods:** This retrospective single-center study included 1060 patients aged ≥60 years: 165 KT recipients and 895 dialysis patients. Propensity score matching using five covariates (age, sex, cardiac disease, cerebrovascular accident, and hemodialysis duration) created balanced cohorts of 123 patients per group. Kaplan–Meier analysis and multivariate Cox regression were performed in the matched cohort, and a time-dependent Cox model was additionally applied to the full cohort to address immortal time bias. **Results:** In the propensity score-matched cohort, KT (HR = 2.72, *p* = 0.009), age (HR = 1.13, *p* < 0.001), and CVD morbidity (HR = 3.84, *p* < 0.001) were independent predictors of mortality. In the time-dependent Cox analysis, KT was not significantly associated with overall survival (HR = 0.94, *p* = 0.837), but a significant KT × CVD interaction was identified (HR = 3.34, *p* = 0.025): KT was associated with reduced mortality in patients without CVD (HR = 0.47, *p* = 0.121) and increased mortality in those with CVD (HR = 1.67, *p* = 0.174). In patients aged ≥65 years with CVD, KT recipients demonstrated significantly worse survival than dialysis patients (*p* = 0.004). **Conclusions:** After correcting for immortal time bias, KT was not significantly associated with overall survival in elderly patients. However, the significant KT × CVD interaction suggests that CVD status is a critical determinant of transplant outcomes.

## 1. Introduction

The global incidence of end-stage renal disease (ESRD) and its associated burden are increasing rapidly [1,2]. This increase is particularly notable among elderly patients. According to the US Renal Data System 2022 Annual Data Report, nearly half of all patients with new ESRD worldwide are elderly patients [2]. South Korea, which is experiencing one of the most rapidly aging populations, noticeably reflects this trend. Among patients with ESRD in South Korea, the proportion of elderly patients has steadily increased from 36.0% in 2010 to 51.9% in 2019 [1]. This demographic shift is significant, as it presents both clinical and ethical challenges in the management of ESRD, particularly regarding the suitability of elderly patients for kidney transplantation (KT).

The number of KTs in elderly patients has been steadily increasing, and the outcomes of KTs are also improving [3,4,5,6,7]. Consequently, KT is considered the preferred treatment option when considering quality of life and longevity for these patients. Multiple studies, including large registry analyses, have demonstrated that KT provides a significant long-term survival benefit over dialysis even in elderly recipients [3,4,5,6,7]. However, recipient age remains a significant factor impacting post-KT mortality rates and graft survival reduction [3,6,8]. In particular, as age increases and in patients with comorbidities such as diabetes mellitus (DM) and cerebrovascular accidents (CVAs), the safety and efficacy of KT are controversial [6,9,10,11]. Although older patients have a lower survival benefit than younger patients, they can still benefit from KT [12]. Nonetheless, assessing the benefits of KT in elderly patients is challenging, and clear guidelines are lacking [13].

The shortage of donated kidneys for KT has led to an increase in the number of patients on waiting lists, with associated risks of morbidity and mortality while waiting [1,2]. To address this, kidney allocation policies are being re-evaluated to balance efficiency and fairness. In the United States and Europe, policies like ‘young-to-young’ and ‘old-to-old’ allocation are being adopted to optimize the use of available kidneys [7,14,15]. These policies aim to match donor and recipient ages more appropriately to address both the ethical considerations and practical outcomes of KT. According to the Korean Society of Nephrology (KSN) ESRD registry, by the end of 2019, the number of hemodialysis (HD) centers had exceeded 1000, and there were more than 30,000 HD machines available [1]. The increased availability and improved management of dialysis have led to a reduction in the overall mortality rate among patients undergoing HD [1]. Consequently, there is a growing need to compare the outcomes of patients undergoing KT with those of waitlisted patients undergoing dialysis and to focus on elderly individuals with comorbidities who are at an increased risk of complications following KT. The purpose of this study was to compare the patient survival (PS) rates of elderly patients (aged older than 60 years) undergoing KT with those of patients on the waiting list.

## 2. Materials and Methods

### 2.1. Patients

The present study involved a retrospective and observational examination conducted at Asan Medical Center (AMC); a group of individuals aged 60 years and older who received their first deceased donor KT between January 2008 and December 2022 was analyzed. The cohort included patients aged 60 years and older, and only first-time KT recipients were included in this study. Patients who underwent multiple KTs (*n* = 17) as well as those who received multiple organ transplants, including pancreas (*n* = 16), heart (*n* = 4), and liver (*n* = 1) transplants, were excluded from the study. As a result, 165 participants were finally included in the KT group. For the comparative analysis, the waiting list group consisted of 895 patients who were registered on AMC’s KT waiting list during the study period. To minimize selection bias in comparing PS, propensity score matching (PSM) was performed using a one-to-one nearest neighbor matching approach with a caliper width of 0.2 standard deviations of the propensity score. Propensity scores were estimated using logistic regression with the following covariates: age, sex, cardiac disease, cerebrovascular accident, and HD duration. Covariate balance after matching was assessed using standardized mean differences (SMDs), with an SMD of less than 0.1 considered indicative of adequate balance. This process yielded two balanced cohorts for survival analysis, consisting of 123 KT recipients and 123 matched dialysis patients from the waiting list. Approval for the research methodology was provided by the Institutional Review Board at AMC (AMC IRB number: 2023-0391). Given the study’s retrospective nature and its classification as a Level 1 study with minimal risk, the IRB exempted it from the requirement for informed consent. Data collection for this research spanned from 1 June 2022 to 28 September 2023. The ethical standards followed in this study were in accordance with the principles stated in the World Medical Association Declaration of Helsinki.

### 2.2. Immunosuppression

Immunosuppressive treatment conformed to the established protocols of AMC [16]. The choice of induction therapy was based on immunological risk, and either basiliximab, an anti-IL-2 receptor monoclonal antibody, or anti-thymocyte globulin (ATG) was employed. ATG was used in patients exhibiting high panel-reactive antibody (PRA) levels or donor-specific antibodies. Initial maintenance therapy incorporated a regimen of calcineurin inhibitors, corticosteroids, and mycophenolate mofetil. During the early postoperative period, the prescribed target trough levels for tacrolimus and cyclosporine were maintained at 7–10 ng/mL and 100–150 ng/mL, respectively. Cyclosporine was used in instances of tacrolimus intolerance or for those assessed to be at a high risk of infection. After the first postoperative year, these target concentrations for tacrolimus and cyclosporine were gradually lowered to 3–6 ng/mL and 50–100 ng/L, respectively. Steroid therapy was initiated with intraoperative methylprednisolone at a dose of 500 mg, followed by gradual reduction, and most patients were maintained on approximately 4 mg/day of methylprednisolone one year following KT.

### 2.3. Definitions

The time from transplantation to the recipient’s death was defined as PS in the transplant group. The PS of the waiting list group was defined as the time from registration on the KT waiting list to the patient’s death. To evaluate the risk factors for PS, we examined the patients’ history of cardiovascular disease (CVD). CVD was divided into cardiac disease and CVAs. Cardiac disease was defined as having a history of percutaneous coronary intervention (PCI), an ejection fraction (EF) of 50% or less as determined by echocardiography, or the presence of atrial fibrillation (AF) as indicated by electrocardiogram findings. CVA was defined as acute infarction on MRI accompanied by neurological symptoms and a medical record of a previous symptomatic cerebral infarction. In the subgroup analysis, patients with cardiac disease and CVAs were categorized as the CVD group. Extended criteria donors (ECDs) were defined as donors aged 60 years or older or those between 50 and 59 years with at least 2 of the following risk factors: a history of arterial hypertension, serum creatinine levels greater than 1.5 mg/dL, or a cause of death due to a CVA [17]. The Kidney Donor Risk Index (KDRI) and Kidney Donor Profile Index (KDPI) were utilized to assess the risk associated with donor kidney grafts [18].

### 2.4. Statistical Analysis

For statistical analysis, categorical variables were analyzed using the Chi-squared test or Fisher’s exact test as appropriate. For continuous variables, Student’s *t*-test was employed. Kaplan–Meier analysis was used to determine cumulative rates of PS, and differences between groups were evaluated using the log-rank test. Univariate and multivariate Cox proportional hazards regression analyses were performed to identify factors affecting PS, and the results are expressed as hazard ratios (HRs). Variables with a *p*-value less than 0.1 in the univariate analysis were subsequently included in the multivariate model. Statistical computations were performed using SPSS version 18.0 (SPSS Inc., Chicago, IL, USA). To ensure comparability between KT recipients and dialysis patients, propensity scores were estimated using logistic regression (scikit-learn library version 1.4.2), and one-to-one nearest neighbor matching was performed with a caliper of 0.2 standard deviations. To address potential immortal time bias inherent in comparing KT recipients with waitlisted patients, a time-dependent Cox proportional hazards model was additionally employed, in which KT was treated as a time-varying covariate. For KT recipients, the follow-up period was split into a pre-transplant interval (during which the patient was classified as waitlisted) and a post-transplant interval. An interaction term between KT and CVD status was included to evaluate whether the effect of transplantation on survival differed according to CVD status. Kaplan–Meier survival curves were fitted to the matched cohorts to evaluate survival outcomes. Statistical computations, matching, and survival analyses were performed using Python (version 3.11) with the scikit-learn(version 1.4.2) and lifelines libraries(version 0.30.1).

## 3. Results

### 3.1. Patient Demographic and Clinical Characteristics

In this study, 1060 patients were analyzed, which included 165 (15.6%) KT recipients and 895 (84.4%) patients on the waiting list. Patients in the KT group were significantly younger (63.8 ± 3.4 years) than those on the waiting list (65.4 ± 4.4 years; *p* < 0.001). Cardiac disease was observed in 11.1% of the total cohort, with significant differences between the groups (*p* = 0.029); this included a lower incidence of PCI, heart failure (EF < 50%), and AF in the KT group than in the waiting list group. There was no significant difference in the prevalence of CVA between the two groups. In the KT group, 35.2% of patients had DM, and the mean duration of dialysis prior to transplantation was 95.1 ± 68.5 months. The mean PRA class I and II levels were 18.1 ± 27.6 and 15.1 ± 25.7, respectively, with an average HLA mismatch of 3.1 ± 1.9. Among the KT recipients, 91.5% were treated with tacrolimus, while 8.5% received cyclosporine. Induction therapy predominantly consisted of basiliximab (83.6%) and ATG (15.2%). In addition, 53.9% of the transplants involved ECDs, with a mean KDPI of 72.3 ± 25.5 and a mean KDRI of 2.5 ± 13.8 (Table 1).

### 3.2. Risk Factors of PS in Deceased Donor KT Recipients

In the univariate regression analysis, age, DM, cardiac disease, and a history of a CVA were identified as independent risk factors affecting PS. However, factors such as an ECD, the KDPI score, and the KDRI score did not demonstrate a significant impact on PS. After adjusting for confounding variables, the multivariate analysis revealed that age (HR = 1.14, 95% confidence interval (CI): 1.01–1.29, *p* = 0.038), DM (HR = 3.35, 95% CI: 1.11–10.13, *p* = 0.032), AF (HR = 7.24, 95% CI: 2.16–24.34, *p* = 0.001), and CVAs (HR = 6.49, 95% CI: 2.51–16.76, *p* < 0.001) were statistically significant risk factors for PS (Table 2).

### 3.3. Causes of Death in Deceased Donor KT Recipients

Table 3 showed the mortality and causes of death among 165 KT patients. The total number of patients was 165; 118 (71.5%) had no history of CVD, and 47 (28.5%) had a history of CVD. The overall mortality rate was 12.1% and was significantly higher in patients with CVD (29.8%) than in those without CVD (5.1%). The leading cause of death was infection, affecting 13 patients (7.8% of the total cohort), and there was a higher prevalence in the CVD group (*n* = 8, 17.0%) than in the non-CVD group (*n* = 5, 4.2%). Pneumonia was the most common infection, causing deaths in 11 patients (6.7%). Other infectious causes included enteritis (*n* = 1, 0.6%) and cellulitis (*n* = 1, 0.6%). Non-infectious causes were myocardial infarction (*n* = 1, 0.6%), malignancy (*n* = 2, 1.2%), and unknown causes (*n* = 1, 0.6%).

#### Propensity Score Matching

After propensity score matching using five covariates (age, sex, cardiac disease, CVA, and HD duration), 123 KT recipients were successfully matched with 123 waiting list patients. The matching employed a caliper width of 0.2 standard deviations of the propensity score, and 42 KT recipients (25.5%) could not be matched within the caliper. Post-matching covariate balance was assessed using SMDs; all matching covariates achieved SMDs below 0.15, indicating adequate balance between the two groups. There were no significant differences in age (64.2 ± 3.6 vs. 64.7 ± 4.3 years, *p* = 0.277), sex (62.6% vs. 56.9% male, *p* = 0.435), cardiac disease (24.4% vs. 21.1%, *p* = 0.648), CVA (13.8% vs. 11.4%, *p* = 0.701), or HD duration (77.0 ± 47.8 vs. 82.0 ± 42.9 months, *p* = 0.391) between the matched KT and waiting list groups (Table 4).

### 3.4. Overall PS in PSM-Matched Cohort

**Figure 1:**
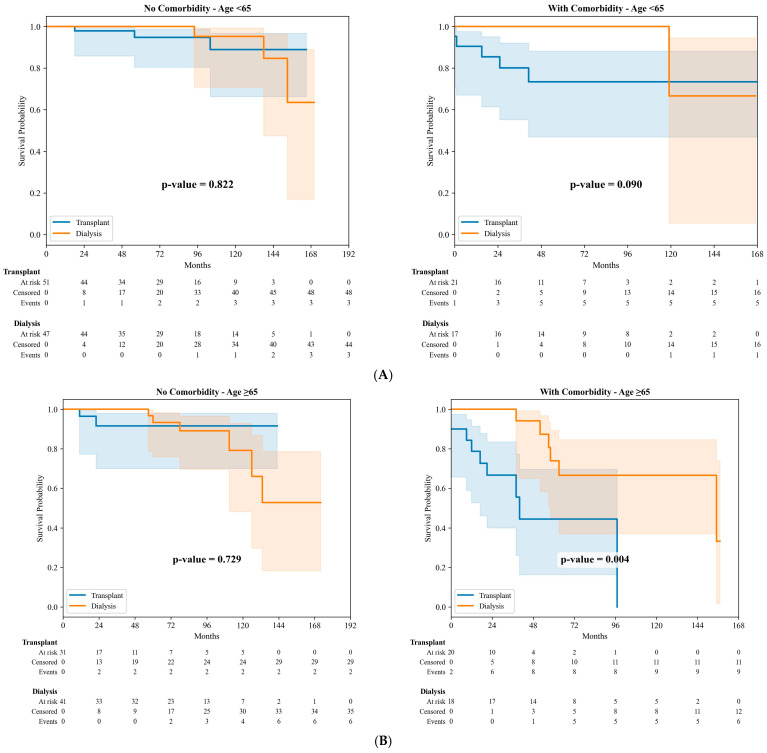
(**A**). Overall patient survival in the propensity score-matched cohort stratified by CVD status in patients aged <65 years. (**B**). Overall patient survival in the propensity score-matched cohort stratified by cardiovascular disease status in patients aged ≥65 years.

In patients younger than 65 years, when stratified by CVD status, there was no significant difference in PS between KT recipients and waiting list patients in either the non-CVD subgroup (*p* = 0.822) or the CVD subgroup (*p* = 0.090), although a trend toward poorer PS was observed among transplant recipients with CVD (Figure 1A).

In patients aged 65 years and older, KT recipients without CVD showed comparable PS to waiting list patients (*p* = 0.729). However, among patients with CVD, KT recipients demonstrated significantly worse PS compared to those on the waiting list (*p* = 0.004) (Figure 1B).

In the univariate Cox regression analysis of the PSM-matched cohort, cardiac disease (HR = 2.32, 95% CI: 1.18–4.58, *p* = 0.015), CVA (HR = 5.91, 95% CI: 2.99–11.66, *p* < 0.001), and CVD morbidity (HR = 4.01, 95% CI: 2.03–7.91, *p* < 0.001) were significantly associated with poorer PS, while KT was not significantly associated with mortality (HR = 1.73, 95% CI: 0.88–3.37, *p* = 0.110). In the multivariate analysis adjusted for age, sex, and CVD morbidity, KT was independently associated with worse PS (HR = 2.72, 95% CI: 1.28–5.77, *p* = 0.009). Age (HR = 1.13, 95% CI: 1.06–1.20, *p* < 0.001) and CVD morbidity (HR = 3.84, 95% CI: 1.90–7.79, *p* < 0.001) were also significant predictors of mortality (Table 5).

#### Time-Dependent Cox Proportional Hazards Analysis

To address potential immortal time bias, a time-dependent Cox model was applied to the full unmatched cohort (*n* = 1050), treating KT as a time-varying covariate. In Model A (main effects), KT was not significantly associated with PS (HR = 0.94, 95% CI: 0.52–1.69, *p* = 0.837), whereas age (HR = 1.09, *p* < 0.001), male sex (HR = 1.51, *p* = 0.019), and cardiac disease (HR = 1.77, *p* < 0.001) were significant predictors.

**Figure 2:**
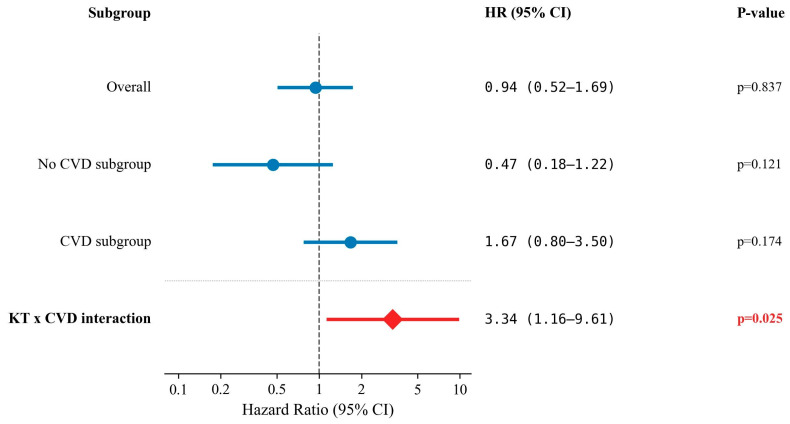
Forest plot of the time-dependent Cox proportional hazards model for patient survival.

In Model B, which included a KT × CVD interaction term, the interaction was statistically significant (HR = 3.34, 95% CI: 1.16–9.61, *p* = 0.025), indicating that the effect of KT on survival differed significantly according to CVD status. In the stratified analysis (Model C), KT was associated with a non-significant reduction in mortality risk in patients without CVD (HR = 0.47, 95% CI: 0.18–1.22, *p* = 0.121), whereas in patients with CVD, KT was associated with a non-significant increase in mortality risk in patients with CVD (HR = 1.67, 95% CI: 0.80–3.50, *p* = 0.174) (Table 6, Figure 2).

## 4. Discussion

This study compared survival outcomes in elderly patients (aged ≥60 years) who underwent deceased donor KT versus those on the waiting list undergoing dialysis. To minimize selection bias, propensity score matching was performed using five covariates (age, sex, cardiac disease, CVA, and HD duration), and a time-dependent Cox model was employed to address immortal time bias by treating KT as a time-varying covariate. After correcting for immortal time bias, KT itself was not significantly associated with overall survival. However, a significant KT × CVD interaction was identified, indicating that the survival effect of KT differed substantially depending on CVD status. These findings underscore the need for personalized treatment strategies for elderly KT candidates, with careful consideration of individual cardiovascular comorbidities when evaluating the expected survival benefit of transplantation.

PS in deceased donor KT varies among studies due to differing characteristics of patient groups, ischemic times, and the status of donor kidneys. Additionally, survival rates among HD patients can differ significantly based on healthcare environment and access to resources. Our study’s five-year PS rate of 82% in the KT group aligns with recently reported outcomes ranging from 65% to 85% in elderly patients [3,9,19,20,21,22,23]. Patients on the waiting list in our study appeared to exhibit a better PS rate of 80% than reported in a recent study among elderly patients on a waiting list, which showed a PS rate of approximately 60% [22,24]. This phenomenon may be attributed to the exclusion of patients with severe health issues or significant comorbidities from the KT registration process, making them ineligible for transplantation [25]. In addition, during the process of preparing and waiting for a KT, pre-assessment and management of risk factors for mortality are performed. As a result, the PS in the waiting list group could have been overestimated when compared with the general dialysis population. In a prior study using U.S. data, patients on dialysis awaiting transplantation had a 38% to 58% lower standardized mortality ratio than all patients undergoing dialysis [12]. Therefore, it is essential to consider these factors when comparing the waiting list group to the KT group in this study. To determine the suitability of deceased donor KT for elderly patients, it is essential to compare the survival rates of HD patients and KT recipients across different healthcare environments and regions.

In general, KT has been reported to provide long-term overall survival benefits, even in elderly patients [4,12,23]. In our study, the time-dependent Cox analysis showed that KT was associated with a non-significant reduction in mortality risk among patients without CVD (HR = 0.47, 95% CI: 0.18–1.22, *p* = 0.121), which is consistent with this reported benefit, although the finding did not reach statistical significance, likely due to the limited sample size. However, in elderly patients undergoing KT, factors such as the recipient’s age, comorbidities, dialysis duration, donor age, and graft condition may have a more pronounced impact on mortality, making these patients more vulnerable to these influences than younger patients [3,8,9,18,21]. As in previous studies, our Cox proportional regression analysis also identified similar risk factors for PS, including age, DM, cardiac disease, and CVAs. However, other conventional risk factors for PS, such as an ECD, the KDPI score, and the KDRI score, did not show significant differences, even in the univariate analysis. This might be because most relatively young patients who have waited long enough to receive grafts from younger donors usually decline to receive kidneys that stray from the standard criteria donor. Therefore, elderly patients tend to receive kidneys from ECDs or older donors. In our study, elderly patients received kidneys with an ECD ratio of over 50%, a KDPI score of 72.3, and a KDRI score of 2.5, which are notably high. This suggests that one of the reasons for lower PS among KT recipients, especially in the CVD group, may be the fact that relatively vulnerable elderly patients received kidneys that deviated from the standard criteria donor.

Considering the increasing rate of KT among the elderly population, it is important to note that these recipients often receive lower-quality kidneys, resulting in inferior survival and graft outcomes [21]. Although rates of death-censored graft loss are similar, elderly recipients face challenges with overall graft survival, mainly due to higher rates of death with a functioning graft [3,21]. This situation sparks a significant debate regarding allocating scarce donor organs, emphasizing the ethical dilemma between maximizing outcomes (utility) and ensuring fairness (equity) [26]. This debate extends to how benefits should be measured, including graft survival, PS, the additional years of life gained through KT, and quality of life. Moreover, it poses disadvantages for patients with lower chances of a favorable outcome, such as older individuals, those with more comorbidities, or those with longer dialysis periods [3,26]. Taking these factors into account, UNOS implemented the New Kidney Allocation System in December 2014, with the goal of enhancing the longevity and allograft-year survival of transplant recipients. This was achieved by giving priority to patients with an estimated post-transplant survival (EPTS) of 20% or lower to receive kidneys from donors with a KDPI score of 20% or lower [14]. While UNOS implemented the “young to young” policy, the Eurotransplant Group introduced the European Senior Program (ESP) alongside its Kidney Allocation System in 1999. The ESP involved establishing a separate waiting list for patients aged 65 years and older and allocating grafts exclusively from donors in the same age group. Patients in the ESP cohort were older with more comorbidities but shorter durations of dialysis. As a result, they experienced significantly better long-term outcomes than the patient cohort before the introduction of this new system [7]. The survival benefit in elderly KT groups is especially prominent in cases of preemptive transplantation and when the KDPI score is low [23]. However, these study results pertain to the entire study population, and whether there is a benefit for personalized risk assessment compared with the waiting list was not evaluated. Recent research on risk evaluation for tailored approaches for each individual has emerged. Chen et al. developed a scoring system in their study, where they scored factors associated with 5-year survival to divide patients into five risk groups. They reported significant differences in PS between the highest and lowest risk score groups, with a 47% five-year mortality for the lowest risk group and over 90% for the highest risk cohort [24]. In addition, Bae et al. developed a tool based on the EPTS and KDPI score, allowing for the direct assessment of risk reduction and its extent between KT recipients and waiting list candidates, contributing to more individualized decision-making [13]. In particular, the survival rates of patients undergoing dialysis are significantly influenced by the healthcare systems in various countries and even within regions of the same country. Therefore, more specific risk assessment tools for dialysis and transplantation that are tailored to each healthcare environment must be developed. In South Korea, the widespread availability of community dialysis centers and the support of national health insurance for providing substantial financial support have led to significant improvements in the survival rates of patients undergoing dialysis, including elderly individuals with DM [1]. Given the current situation, in-depth research on the survival benefits for elderly high-risk patients undergoing KT that considers both PS and the efficient allocation of scarce donor organs must be performed.

Our study has several limitations. First, it was a retrospective study conducted at a single center; thus, the findings may not fully represent the entire population of patients undergoing KT and dialysis. Second, although we performed multivariable propensity score matching using five covariates and applied a time-dependent Cox model to address immortal time bias, important comorbidity data such as diabetes mellitus, hypertension, and body mass index were unavailable for the waiting list group and therefore could not be included in the matching or regression models. This may have resulted in residual confounding that could influence the survival comparison between the two groups. Third, the propensity score matching yielded 123 matched pairs from the original 165 KT recipients, resulting in a reduced sample size that may have limited the statistical power to detect significant differences in subgroup analyses. Fourth, patients not registered on the waiting list immediately after starting dialysis could lead to an overestimation of survival in the waiting list group. Fifth, patients on the waiting list represent a selected population with relatively favorable health status compared with the overall dialysis population, as individuals with severe comorbidities are generally excluded from transplant candidacy. This selection process may have resulted in an overestimation of survival in the dialysis group, and this inherent bias cannot be fully eliminated by statistical adjustment. Additionally, the waiting list group’s survival may have been overestimated compared to both general dialysis patients and other waiting list populations. Fifth, the patients in the waiting list group primarily received dialysis at local centers and did not undergo regular laboratory or physical examinations at our center. Obtaining more detailed information to analyze risk factors was challenging in this group.

## 5. Conclusions

Our study demonstrated that after correcting for immortal time bias using a time-dependent Cox model, KT was not significantly associated with overall survival in elderly patients. However, a significant KT × CVD interaction indicated that the survival effect of KT differed according to cardiovascular disease status: KT showed a favorable outcome in patients without CVD, whereas it was associated with worse outcomes in those with CVD, particularly in patients aged 65 years and older. These findings suggest that cardiovascular comorbidity should be a central consideration in the decision-making process for elderly KT candidates. Therefore, it is crucial for each transplant center to make efforts to develop tools for assessing their own transplant survival rates and the risk profiles of patients in their local community undergoing dialysis. These efforts can facilitate a more individualized and objective risk assessment, ultimately contributing to increased survival rates among elderly patients and the efficient allocation of scarce donor kidneys.

## Figures and Tables

**Table 1:** Baseline and clinical characteristics.

	Total	KT	Waiting List	*p*-Value
**Number of patients**	1060 (100)	165 (15.6)	895 (84.4)	-
Mean age (years)	65.1 ± 4.2	63.8 ± 3.4	65.4 ± 4.4	<0.001
Female sex	644 (60.8)	101 (61.2)	543 (60.7)	0.89
Cardiac disease				0.029
PCI	118 (11.1)	14 (8.5)	104 (11.6)	
Heart failure (EF < 50%)	82 (7.7)	8 (4.8)	74 (8.3)	
Atrial fibrillation	107 (10.1)	10 (6.1)	97 (10.8)	
CVA	127 (12.0)	22(13.3)	105 (11.7)	0.56
**Transplantation characteristics**				
Diabetes mellitus		58 (35.2)		N/A
Dialysis duration		95.1 ± 68.5		N/A
PRA class I		18.1 ± 27.6		N/A
PRA class II		15.1 ± 25.7		N/A
HLA mismatch		3.1 ± 1.9		N/A
Calcineurin inhibitor				N/A
Tacrolimus		151 (91.5)		
Cyclosporin		14 (8.5)		
Induction				N/A
Basiliximab		138 (83.6)		
Anti-thymocyte globulin		25 (15.2)		
ECD donation		89 (53.9)		N/A
Donor KDPI		72.3 ± 25.5		N/A
Donor KDRI		2.5 ± 13.8		N/A

Continuous data are presented as means ± standard deviations. Categorical data are presented as a number (%). Abbreviations: KT, kidney transplantation; PCI, percutaneous coronary intervention; EF, ejection fraction; CVA, cerebrovascular accident; PRA, panel reactive antibody; HLA, human leukocyte antigen; ECD, extended criteria donor; KDPI, kidney donor risk factor; KDRI, kidney donor risk index; N/A, not applicable.

**Table 2:** Risk factors for patient survival following kidney transplantation.

	Univariate Analysis		Multivariate Analysis	
	HR (95% CI)	*p*-Value	HR (95% CI)	*p*-Value
Age	1.22 (1.10–1.36)	<0.001	1.14 (1.01–1.29)	0.038
Male sex	1.98 (0.72–5.45)	0.19		
PRA class I	0.99 (0.97–1.01)	0.21		
PRA class II	0.99 (0.97–1.01)	0.37		
Dialysis duration	1.00 (0.99–1.01)	0.93		
HLA mismatch	0.93 (0.74–1.16)	0.51		
Diabetes mellitus	2.89 (1.18–6.95)	0.021	3.35 (1.11–10.13)	0.032
Cardiac disease		0.001		0.028
PCI	1.72 (0.38–7.70)	0.48	3.57 (0.70–18.22)	0.13
Heart failure	1.43 (0.19–11.02)	0.73	1.31 (0.14–11.94)	0.81
Atrial fibrillation	8.42 (2.92–24.27)	<0.001	7.24 (2.16–24.34)	0.001
CVA	10.71 (4.39–26.13)	<0.001	6.49 (2.51–16.76)	<0.001
ECD donation	1.10 (0.45–2.65)	0.84		
Donor KDPI	1.00 (0.99–1.02)	0.71		
Donor KDRI	0.99 (0.77–1.24)	0.84		

Abbreviations: PRA, panel reactive antibody; HLA, human leukocyte antigen; PCI, percutaneous coronary intervention; CVA, cerebrovascular accident; ECD, extended criteria donor; KDPI, kidney donor risk factor; KDRI, kidney donor risk index.

**Table 3:** Causes of death in deceased donor kidney transplant patients.

	Total	Without CVD	With CVD
**Number of patients**	165 (100)	118 (71.5)	47 (28.5)
**Mortality**	20 (12.1)	6 (5.1)	14 (29.8)
**Cause**			
Infection	13 (7.8)	5 (4.2)	8 (17.0)
Pneumonia	11 (6.7)	4 (3.4)	7 (14.9)
Enteritis	1 (0.6)		1 (2.1)
Cellulitis	1 (0.6)	1 (0.8)	
Myocardial infarction	1 (0.6)		1 (2.1)
Malignancy	2 (1.2)	1 (0.8)	1 (2.1)
Ischemic bowel disease	3 (1.8)		3 (6.4)
Unknown	1 (0.6)		1 (2.1)

Categorical data are presented as a number (%).

**Table 4:** Baseline and clinical characteristics after propensity score matching.

Variable	KT (*n* = 123)	WL (*n* = 123)	*p*-Value	SMD
Age (years)	64.2 ± 3.6	64.7 ± 4.3	0.277	0.139
Male sex	77 (62.6%)	70 (56.9%)	0.435	0.116
Cardiac disease	30 (24.4%)	26 (21.1%)	0.648	0.077
PCI	13 (10.6%)	9 (7.3%)	0.503	0.114
HF (EF < 50%)	7 (5.7%)	5 (4.1%)	0.767	0.075
AF	10 (8.1%)	12 (9.8%)	0.823	0.057
CVA	17 (13.8%)	14 (11.4%)	0.701	0.073
CVD (cardiac + CVA)	41 (33.3%)	35 (28.5%)	0.490	0.105
HD duration (months)	77.0 ± 47.8	82.0 ± 42.9	0.391	0.110

Continuous data are presented as means ± standard deviations. Categorical data are presented as number (%). Propensity score matching was performed using age, sex, cardiac disease, CVA, and hemodialysis duration. SMD, standardized mean difference; KT, kidney transplantation; WL, waiting list; PCI, percutaneous coronary intervention; EF, ejection fraction; AF, atrial fibrillation; CVA, cerebrovascular accident; CVD, cardiovascular disease; HD, hemodialysis.

**Table 5:** Risk factors for patient survival in propensity score-matched cohort (*n* = 246).

Variable	Univariate HR (95% CI)	*p*-Value	Multivariate HR (95% CI)	*p*-Value
KT (vs. dialysis)	1.73 (0.88–3.37)	0.110	2.72 (1.28–5.77)	0.009
Age (per year)	1.12 (1.06–1.18)	<0.001	1.13 (1.06–1.20)	<0.001
Male sex	1.77 (0.86–3.62)	0.121	1.05 (0.49–2.24)	0.897
Cardiac disease	2.32 (1.18–4.58)	0.015		
CVA	5.91 (2.99–11.66)	<0.001		
Morbidity (CVD)	4.01 (2.03–7.91)	<0.001	3.84 (1.90–7.79)	<0.001

Propensity score matching was performed using age, sex, cardiac disease, CVA, and hemodialysis duration with a caliper of 0.2 standard deviations. Morbidity = cerebrovascular disease or cardiac disease. HR, hazard ratio; CI, confidence interval; KT, kidney transplantation; CVA, cerebrovascular accident; CVD, cardiovascular disease.

**Table 6:** Time-dependent Cox proportional hazards model for patient survival with kidney transplantation as a time-varying covariate.

Variable	Model A HR (95% CI)	*p*-Value	Model B HR (95% CI)	*p*-Value	Model C HR (95% CI)	*p*-Value
KT (time-varying)	0.94 (0.52–1.69)	0.837	0.50 (0.20–1.22)	0.126		
Age (per year)	1.09 (1.06–1.12)	<0.001	1.09 (1.06–1.12)	<0.001		
Male sex	1.51 (1.07–2.14)	0.019	1.50 (1.06–2.12)	0.023		
Cardiac disease	1.77 (1.28–2.46)	<0.001	1.38 (0.69–2.75)	0.359		
CVA	1.48 (1.00–2.19)	0.052	1.24 (0.73–2.10)	0.429		
CVD			1.23 (0.56–2.67)	0.610		
KT × CVD interaction			3.34 (1.16–9.61)	0.025		
**No CVD subgroup (** ***n* = 667)**						
KT (time-varying)					0.47 (0.18–1.22)	0.121
Age (per year)					1.12 (1.07–1.18)	<0.001
Male sex					1.77 (1.07–2.93)	0.025
**CVD subgroup (** ***n* = 383)**						
KT (time-varying)					1.67 (0.80–3.50)	0.174
Age (per year)					1.08 (1.04–1.12)	<0.001
Male sex					1.40 (0.86–2.27)	0.175

Full unmatched cohort (*n* = 1050) with kidney transplantation modeled as a time-varying covariate to address immortal time bias. Model A: main effects only. Model B: includes KT × CVD interaction term. Model C: stratified analysis by CVD status. HR, hazard ratio; CI, confidence interval; KT, kidney transplantation; CVA, cerebrovascular accident; CVD, cardiovascular disease.

## Data Availability

The raw data supporting this study’s findings are available from the corresponding author on reasonable request, without undue delay or restriction.
